# Properties of Contextual Memory Formed in the Absence of αCaMKII Autophosphorylation

**DOI:** 10.1186/1756-6606-4-8

**Published:** 2011-01-28

**Authors:** Elaine E Irvine, Arthur Danhiez, Kasia Radwanska, Charlotte Nassim, Walter Lucchesi, Emile Godaux, Laurence Ris, K Peter Giese

**Affiliations:** 1 Wolfson Institute for Biomedical Research, University College London, Gower Street, London, WC1E 6BT, UK; 2 Metabolic Signalling Group, MRC Clinical Sciences Centre, Imperial College London, Du Cane Road, London, W12 0NN, UK; 3 Department of Neurosciences, University of Mons, 20 Place du Parc, B-7000 Mons, Belgium; 4 Centre for the Cellular Basis of Behaviour, Institute of Psychiatry, King's College London, 125 Coldharbour Lane, London, SE5 9NU, UK; 5 Laboratory of Molecular Neurobiology, Nencki Institute, Warsaw, Poland

## Abstract

The alpha-isoform of calcium/calmodulin-dependent kinase II (αCaMKII) is a major synaptic kinase that undergoes autophosphorylation after NMDA receptor activation, switching the kinase into a calcium-independent activity state. This αCaMKII autophosphorylation is essential for NMDA receptor-dependent long-term potentiation (LTP), induced by a single tetanus, in hippocampal area CA1 and in neocortex. Furthermore, the αCaMKII autophosphorylation is essential for contextual long-term memory (LTM) formation after a single training trial but not after a massed training session. Here, we show that in the absence of αCaMKII autophosphorylation contextual fear conditioning is hippocampus dependent and that multi-tetanus-dependent late-LTP cannot be induced in hippocampal area CA1. Furthermore, we show that in the absence of αCaMKII autophosphorylation contextual LTM persists for 30 days, the latest time point tested. Additionally, contextual, but not cued, LTM formation in the absence of αCaMKII autophosphorylation appears to be impaired in 18 month-old mice. Taken together, our findings suggest that αCaMKII autophosphorylation-independent plasticity in the hippocampus is sufficient for contextual LTM formation and that αCaMKII autophosphorylation may be important for delaying age-related impairments in hippocampal memory formation. Furthermore, they propose that NMDA receptor-dependent LTP in hippocampal area CA1 is essential for contextual LTM formation after a single trial but not after massed training. Finally, our results challenge the proposal that NMDA receptor-dependent LTP in neocortex is required for remote contextual LTM.

## Background

A major goal in neuroscience is to understand the molecular and cellular mechanisms underlying learning and memory. Synaptic plasticity, in particular NMDA receptor-dependent long-term potentiation (LTP), is thought to be an important cellular mechanism of memory formation that can be induced by behavioral training [1-4]. An essential signaling molecule downstream of NMDA receptor activation is the alpha-isoform of calcium/calmodulin-dependent protein kinase II (αCaMKII), the major post-synaptic density protein in the hippocampus [5]. After activation αCaMKII can autophosphorylate at threonine-286 (T286), switching the kinase into a calcium/calmodulin-independent, autonomous activity state. This T286 autophosphorylation is essential for NMDA receptor-dependent LTP at excitatory synapses in hippocampal area CA1 and neocortex, as indicated by studies with αCaMKII autophosphorylation-deficient knockin (αCaMKII^T286A^) mice [6-9].

Hippocampal area CA1 is essential for memory formation after contextual fear conditioning, a task in which a rodent learns to associate a neutral environment (context) with an aversive foot shock [10,11]. The αCaMKII^T286A ^mutant mice have impaired contextual fear long-term memory (LTM) formation after a single training trial or a massed session of 3 trials [12]. However, unexpectedly αCaMKII^T286A ^mutants can form contextual LTM after a massed training session of 5 trials [12]. This finding posed several mechanistic questions, which we have addressed here. We studied whether a) short-term memory (STM) formation depends on αCaMKII autophosphorylation in the same way as LTM formation, b) spaced training also enables contextual LTM formation without αCaMKII autophosphorylation, c) contextual LTM formation in the absence of αCaMKII autophosphorylation requires the hippocampus, d) multiple tetani can induce L-LTP in hippocampal area CA1 in the absence of αCaMKII autophosphorylation, e) remote contextual LTM requires αCaMKII autophosphorylation, f) LTM formation in the absence of αCaMKII autophosphorylation is more sensitive to aging than LTM formation with intact αCaMKII autophosphorylation.

## Results

### αCaMKII autophosphorylation is necessary for contextual STM formation after 3, but not 5, tone-shock pairings

There is evidence to suggest that STM may be an independent process to LTM [13]. Our previous studies have shown that αCaMKII autophosphorlyation is essential for contextual LTM formation after training with either 1 or 3 tone-shock pairings but is not needed after a more intense training protocol of 5 tone-shock pairings [12]. Therefore, we wanted to investigate whether the αCaMKII^T286A ^mice have the same contextual STM phenotype as that observed for contextual LTM.

**Figure 1:**
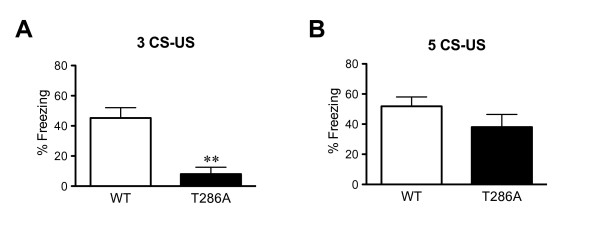
**Impaired contextual STM formation after 3, but not 5, conditioning trials in αCaMKII autophosphorylation-deficient mice**. Means ± s.e.m. are shown. **a**: WT mice (n = 6) and αCaMKII^T286A ^mutants (n = 6) were trained with 3 tone-shock pairings and tested for contextual STM 2 hours after conditioning. The mutants were significantly impaired. **, P < 0.01. **b**: WT mice (n = 10) and αCaMKII^T286A ^mutants (n = 10) were trained with 5 tone-shock pairings and tested for contextual STM 2 hours after conditioning. The freezing response did not differ between the genotypes.

Mice were trained with either 3 or 5 tone-shock pairings and then tested 2 h later to assess their contextual STM. After training with 3 tone-shock pairings the WT mice formed a contextual STM (Figure 1a). However, the αCaMKII^T286A ^mutants did not show significant freezing to the conditioning context and were significantly impaired compared to their WT littermates (H = 8.4, P = 0.002; Figure 1a). Another set of naïve mice was then subjected to a more intensive conditioning protocol with 5 tone-shock pairings. After this training protocol not only WT mice, but also the αCaMKII^T286A ^mutants were able to form contextual STM and the freezing levels were not different between the two genotypes (F_(1,18) _= 0.77, P = 0.39; Figure 1b). In summary, like contextual LTM formation, contextual STM formation is impaired by the lack of αCaMKII autophosphorylation after 3, but not 5, conditioning trials.

### Spaced training does not enable contextual LTM formation in the absence of αCaMKII autophosphorylation

**Figure 2:**
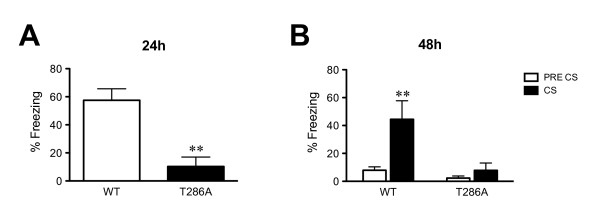
**Contextual LTM formation after 2 one-trial conditioning trials with an inter-trial interval of 1 h is impaired in αCaMKII autophosphorylation-deficient mice**. Means ± s.e.m. are shown. **a**: WT mice (n = 6) and αCaMKII^T286A ^mutants (n = 6) were trained twice with a single CS-US pairing (inter-trial interval of 1 h), and then tested 24 h and 48 h after conditioning for contextual and cued LTM formation respectively. The αCaMKII^T286A ^mutants had impaired contextual LTM formation as they showed significantly less freezing than their WT littermates. **b**: Forty-eight hours after training cued fear LTM was impaired as the αCaMKII^T286A ^mutants did not freeze to the tone when tested 48h after conditioning. **, P < 0.01.

Mice lacking the alpha- and delta-isoform of the transcription factor cAMP-responsive element binding protein (CREB) are impaired in contextual LTM formation after a single training trial or massed training session with 5 trials [14,15]. However, 2 spaced training trials (1 h inter-trial interval) enable contextual LTM formation in these CREB mutants [15]. Accordingly it has been argued that spaced training is more efficient for LTM formation than massed training [16]. We tested this proposal with the αCaMKII^T286A ^mutants using the same training protocol that is sufficient for contextual LTM formation in the CREB^αΔ ^mutants [15] (Figure 2). Mice were trained twice with a single CS-US pairing (1 h inter-trial interval) and then tested 24 h and 48 h later for contextual and cued LTM formation respectively. Twenty-four hours after conditioning the αCaMKII^T286A ^mutants had significantly impaired contextual LTM formation as they froze significantly less than their WT littermates (F_(1,12) _= 4.4, P = 0.005; Figure 2a). Furthermore, the αCaMKII^T286A ^mutants had impaired cued fear conditioning as they did not freeze when the tone was presented during the test 48 h after training, whereas their WT littermates had a significant increase in freezing when the tone was presented (interaction, F_(1,10) _= 5.7, P < 0.05; effect of genotype, F_(1,10) _= 6.8, P < 0.05 and effect of tone, F_(1,10) _= 10.6, P < 0.005; Figure 2b). Therefore, spaced training was not sufficient for fear LTM formation in the αCaMKII^T286A ^mutants.

### Contextual LTM formation in the absence of αCaMKII autophosphorylation is hippocampus-dependent

**Figure 3:**
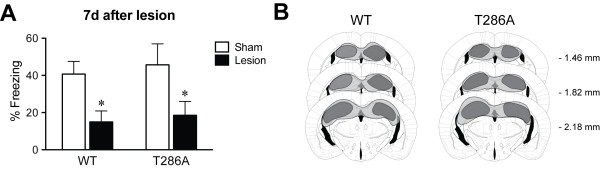
**Post-training lesions of the dorsal hippocampus impair contextual fear LTM formation after 5 conditioning trials in αCaMKII autophosphorylation-deficient mice**. Means ± s.e.m. are shown. **a**: Twenty-four hours after conditioning WT mice (Sham, n = 14 and Lesioned, n = 8) and αCaMKII^T286A ^mutants (Sham, n = 12 and Lesioned, n = 8) were sham treated or the dorsal hippocampus was lesioned. Seven days after lesioning the mice were tested for contextual fear LTM. There was no significant difference between the genotypes, but the hippocampal lesion significantly impaired the contextual LTM in both genotypes. **b**: Histological assessment of the dorsal hippocampal lesions for both genotypes. *, P < 0.05.

In WT mice background contextual fear conditioning is hippocampus-dependent. This has been demonstrated with post-training lesions, whilst pre-training lesions allow for compensation mechanisms to come into play that enable learning [17,18]. Because in the αCaMKII^T286A ^mutants one tetanus-induced NMDA receptor-dependent LTP in hippocampal area CA1 is totally abolished [6,8,9] it was conceivable that the contextual LTM formation in the mutants after 5 training trials [12] is not hippocampus-dependent but rather requires another brain area due to systems compensation. If this were the case, then this would have implications for understanding the role of NMDA receptor-dependent LTP in memory formation. Therefore, to study if contextual fear conditioning in the αCaMKII^T286A ^mutants requires the hippocampus we performed a post-training lesion experiment. Twenty-four hours after 5 tone-shock pairings the dorsal hippocampus was lesioned with ibotenic acid in αCaMKII^T286A ^mutants and their WT littermates (Figure 3a). Seven days after lesioning the mice were tested for contextual LTM. Sham-treated animals were used as controls. We found that hippocampal lesioned mice of both genotypes had impaired contextual LTM formation (effect of lesion, F_(1,38) _= 8.6, P = 0.006, but no effect of genotype F_(1,38) _= 0.2, NS or interaction F_(1,38) _= 0.006, NS; Figure 3b) showing that contextual fear conditioning in the absence of αCaMKII autophosphorylation is hippocampus-dependent.

### Multi-tetanus-dependent late-LTP in hippocampal area CA1 cannot be induced in the absence of αCaMKII autophosphorylation

**Figure 4:**
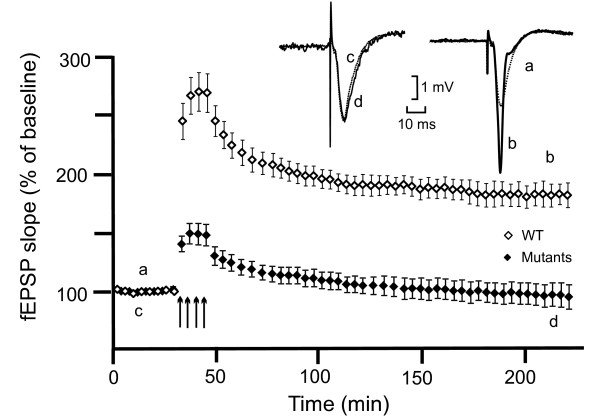
**CA1 L-LTP is absent in αCaMKII autophosphorylation-deficient mice**. fEPSP slope compared to baseline. Means ± s.e.m. are shown. Four trains of high frequency stimulation induced in hippocampal area CA1 an L-LTP in WT mice (n = 6, open symbols) and an early potentiation that declined to baseline within one hour in αCaMKII^T286A ^mutants (Mutants, n = 6, filled symbols). Traces show representative recordings of fEPSP before and three hours after the trains in mutants (left: c,d) and in WT (right: a,b).

The lesion result together with previous CA1 LTP recordings using a single tetanus [6,8,9] suggested that NMDA receptor-dependent LTP in hippocampal area CA1, which is impaired in the absence of αCaMKII autophosphorylation, is not essential for contextual LTM formation. However, it remained possible that in the absence of αCaMKII autophosphorylation multi-tetanus-dependent late-LTP (L-LTP) in hippocampal area CA1 may be induced paralleling contextual LTM formation that occurs only after several training trials [12]. Therefore, we studied the induction of L-LTP in area CA1 using extracellular field recordings in hippocampal slices (Figure 4). In agreement with previous hippocampal slice recordings we found that basal synaptic transmission did not differ between the genotypes (data not shown), in agreement with previous hippocampal slice recordings [6,8,9]. In WT mice (n = 6) L-LTP was obtained as stimulation with 4 tetani induced an increase in the slope of the field excitatory postsynaptic potential (fEPSP) slope to 246 ± 16%, that was maintained at 178 ± 14% 3 hours later. L-LTP was not obtained in αCaMKII^T286A ^mutants (n = 6) although there was a transient potentiation. After the tetani the response potentiated at 140 ± 6% but returned to baseline within one hour. The difference between the genotypes was statistically significant (two-way ANOVA, F_(1,48) _= 23.97, p < 0.0001, Student-Newman-Keuls Method, p < 0.05) at every point after the tetani (Student t test p < 0.05). Thus, CA1 L-LTP was not induced in the absence of αCaMKII autophosphorylation after multiple tetani.

### αCaMKII autophosphorylation is not necessary for remote contextual LTM

In normal rodents contextual LTM becomes hippocampus-independent over a period of approximately 30 days [19,20]. Studies with heterozygous αCaMKII null mutants have suggested that neocortical NMDA receptor-dependent LTP is essential for the reorganization of contextual LTM [21]. We were able to test this hypothesis with the αCaMKII^T286A ^mutants as these animals form contextual LTM 24 hours after 5 conditioning trials [12] and because they have fully blocked NMDA receptor-dependent LTP in neocortex [7].

**Figure 5:**
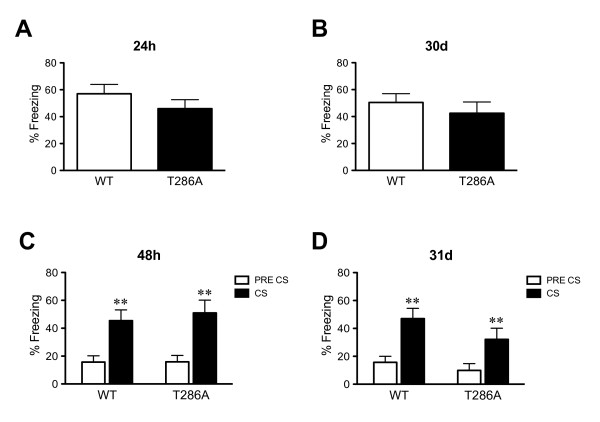
**Normal remote contextual and cued fear LTM after five conditioning trials in αCaMKII autophosphorylation-deficient mice**. Means ± s.e.m. are shown. **a**: WT mice (n = 14) and αCaMKII^T286A ^mutants (n = 14) were trained with five tone-shock pairings and tested for recent contextual LTM 24 hours after conditioning. Freezing did not differ between the genotypes. **b**: Thirty days after training the αCaMKII^T286A ^mutants had normal remote contextual LTM as freezing did not differ between the genotypes, suggesting that the contextual LTM was stable. **c**: Forty-eight hours after training cued fear LTM was normal as both genotypes showed a similar significant increase in freezing when the tone was presented. **d**: The cued fear LTM was stable as 31 days after training mice of both genotypes still showed a similar significant increase in freezing when the tone was presented. **, P < 0.01.

Firstly, we confirmed our earlier finding that the αCaMKII^T286A ^mutants are able to form both contextual and cued fear LTM as they had similar freezing levels to their WT littermates when tested 24 and 48 hours, respectively, after 5 tone-shock pairings (Context, F_(1,26) _= 1.32, P = 0.26; Tone, effect of tone, F_(1,26) _= 60.7, p < 0.001, but no effect of genotype F_(1,26) _= 0.1 or interaction F_(1,26) _= 0.4; Figure 5a and 5c). These mice were then tested again 30 and 31 days after training for remote contextual and cued fear LTM (Figure 5b and 5d). We found that αCaMKII^T286A ^mutants and WT littermates had an equal amount of contextual freezing when tested 30 days after training (H = 0.2, P = 0.65; Figure 5b). Furthermore, as previously shown the αCaMKII^T286A ^mutants had normal remote cued fear LTM as both genotypes froze significantly more during tone presentation (effect of tone, F_(1,26) _= 32.1, p < 0.001, but no effect of genotype F_(1,26) _= 1.8 or interaction F_(1,26) _= 0.9; Figure 5d). Taken together, these results show that αCaMKII autophosphorylation is not required for remote contextual LTM after overtraining and questions whether neocortical NMDA receptor-dependent LTP is in fact essential for the reorganization of contextual LTM as previously suggested [21].

### Contextual LTM formation in the absence of αCaMKII autophosphorylation is impaired at 18 months of age

**Figure 6:**
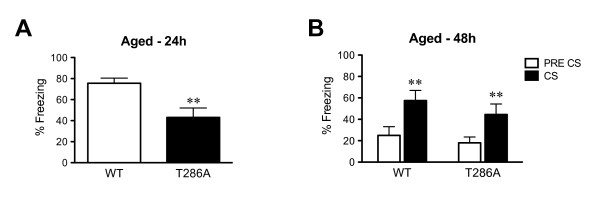
**Contextual LTM formation after 5 conditioning trials is impaired in 18 month-old αCaMKII autophosphorylation-deficient mice**. Means ± s.e.m. are shown. **a**: Eighteen-month-old WT mice (n = 10) and αCaMKII^T286A ^mutants (n = 10) were trained with five tone-shock pairings and tested for recent contextual LTM 24 hours after conditioning. The mutants showed significantly less freezing. **b**: The same mice as in **a **were tested for cued fear memory 24 hours after the context test. For both genotypes there was significant tone fear memory. **, P < 0.01.

Changes in molecular signaling in the hippocampus can impact on age-dependence of contextual LTM formation [e.g., [22]]. Therefore, we studied whether contextual LTM formation after 5 tone-shock pairings was altered in aged αCaMKII^T286A ^mutants (18 months old) (Figure 6a). Twenty-four hours after conditioning the aged αCaMKII^T286A ^mutants had significantly reduced contextual freezing indicating impaired contextual LTM formation (F_(1,19) _= 9.95, P = 0.005). This impairment in contextual LTM formation is unlikely to have resulted from deficits in amygdala function in the aged αCaMKII^T286A ^mutants as they had normal cued fear LTM 48 hours after training (effect of tone, F_(1,18) _= 35.1, p < 0.001, but no effect of genotype F_(1,18) _= 0.9 or interaction F_(1,18) _= 0.4, Figure 6b). It should be noted that the contextual freezing levels of the aged WT mice was higher than for young adult WT mice (Figure 5a). Since contextual fear conditioning was done in distinct cohorts for the different ages, our findings only suggest an age-dependent impairment in contextual fear conditioning of the αCaMKII^T286A ^mutants. Nonetheless, our results propose that αCaMKII autophosphorylation is important for delaying age-related memory impairments.

## Discussion

Here, we have used the αCaMKII^T286A ^mutant mice to investigate several mechanistic questions of contextual LTM formation. Firstly, we have shown that like contextual LTM formation, contextual STM formation is impaired in the absence of αCaMKII autophosphorylation after 3, but not 5, conditioning trials. In principle, this result is consistent with the idea that STM may be a prerequisite for LTM formation. However, in earlier studies we found that αCaMKII autophosphorylation is required for the induction of immediate-early gene (IEG) expression by contextual fear conditioning [[23]; Radwanska et al., unpublished data]. IEG expression, which is essential for LTM formation [e.g., [24]], is induced 'immediately' after training. Therefore, it seems more likely that STM and LTM formation occur in parallel as independent processes as suggested by Izquierdo and colleagues [13]. In this case αCaMKII autophosphorylation is needed for both types of memory formation by triggering distinct molecular processes. However, we did not observe a genetic dissociation between STM and LTM formation indicating that αCaMKII autophosphorylation is required for both.

We found that massed, but not spaced, training enables contextual LTM formation in αCaMKII^T286A ^mutants. This is the opposite to contextual LTM formation in CREB^αΔ-/- ^mutants that is enabled by spaced, but not massed, training [15]. It is not known why spaced training enables LTM formation in the CREB hypomorphic mutants. In this case, a process needs to be 'remembered' at the second training trial one hour after the first trial. Consequently, the activation of such a process seems to require the αCaMKII autophosphorylation. However, the nature of the process remains unclear. The comparison of the contextual LTM phenotypes in the CREB mutants and the αCaMKII^T286A ^mutants shows that spaced training is not always more beneficial than massed training for LTM formation. It suggests that massed training may be more beneficial for LTM formation when synaptic signaling is impaired, whereas spaced training may be more beneficial for LTM formation when transcription in the nucleus is affected.

We have shown that contextual fear conditioning in the αCaMKII^T286A ^mutants is hippocampus dependent and that multi-tetanus-dependent L-LTP cannot be induced in hippocampal area CA1 in these mice. Together, with previous hippocampal slice recordings [6,8,9] this strongly suggests that in the absence of αCaMKII autophosphorylation there is no hippocampal CA1 NMDA receptor-dependent LTP and therefore that contextual LTM does not necessarily require this type of plasticity. This is an unexpected proposal because an earlier study showed that CA1-restricted knockout of the essential NMDA receptor subunit NR1 impairs contextual LTM formation [10]. However, Rampon et al. investigated contextual LTM formation after a single training trial [10]. Consistently, the absence of αCaMKII autophosphorylation blocks contextual LTM formation after a single training trial [12]. Thus, we suggest that CA1 NMDA receptor-dependent LTP has a specific role in contextual fear conditioning after one trial, but it is not required for LTM formation after 5 massed trials.

We also found that αCaMKII autophosphorylation is not required for remote contextual LTM after massed training. As NMDA receptor-dependent LTP in the neocortex is blocked in the αCaMKII^T286A ^mutants, the finding of normal remote contextual LTM in these animals suggests that NMDA receptor-dependent LTP in neocortex is not required for the reorganization of contextual LTM. This suggestion is in contrast with an earlier suggestion from a study with heterozygous αCaMKII null mutants that have impaired NMDA receptor-dependent LTP in neocortex [21]. However, these heterozygous null mutants also have altered neurogenesis in the adult dentate gyrus [25], which has recently been implicated in regulating the reorganization of contextual LTM [26]. Consistently, the αCaMKII^T286A ^mutants have normal neurogenesis in adult dentate gyrus [27] and normal remote contextual LTM. It should be noted that our findings do not claim that αCaMKII is dispensable for remote contextual LTM. This is because αCaMKII has various functions and only a subset of these functions is impaired in the αCaMKII^T286A ^mutants. For example, αCaMKII has a structural function that is independent from the T286 autophosphorylation [28]. Thus, it is not surprising that heterozygous αCaMKII null mutants, but not αCaMKII^T286A ^mutants, have impaired remote contextual LTM.

Finally, we provide some evidence that 18 month-old αCaMKII^T286A ^mutants are impaired in contextual, but not cued, LTM formation. However, more systematic aging studies are required to further establish whether aged αCaMKII^T286A ^mutants have impaired contextual LTM formation. Nonetheless, our preliminary findings suggest that αCaMKII autophosphorylation-dependent plasticity in the hippocampus is important to delay age-dependent memory impairments. This idea may be of relevance for understanding Alzheimer's disease that has been suggested to be associated with impaired αCaMKII autophosphorylation [29-31].

## Conclusions

One of the main conclusions from our studies is that contextual fear conditioning in the absence of αCaMKII autophosphorylation is hippocampus-dependent. Thus, hippocampal plasticities that do not require αCaMKII autophosphorylation appear to be sufficient for contextual LTM formation. Further, NMDA receptor-dependent LTP in hippocampal area CA1 that requires αCaMKII autophosphorylation seems to have a specific role in contextual LTM formation: it is needed for one-trial LTM formation but not for LTM formation after a session of massed training trials. Additionally, our findings suggest that NMDA receptor dependent LTP in neocortex is not required for reorganization of contextual LTM as previously proposed [21], as remote contextual LTM is intact in the absence of αCaMKII autophosphorylation and indeed autophosphorylation is essential for this type of LTP [7]. Finally, we suggest that αCaMKII autophosphorylation may be important for delaying age-related hippocampus-dependent memory decline.

## Methods

### Animals

The subjects were housed in groups of two to five and maintained on a 12 h light/dark cycle with food and water *ad libitum*. Male and female homozygous αCaMKII^T286A ^mutants and control wild-type (WT) littermates were obtained in the 129B6F4-6 background by intercrossing heterozygous mutants. Genotyping was carried out by PCR analysis, as described previously [6] with DNA obtained from tail biopsies on postnatal day 21, the day of weaning. All the young, adult mice were 10-16 weeks of age, and all aged mice were 18 months old, at the time of training. All experiments used approximately equal numbers of male and female mice, and were undertaken in accordance with the UK Animals (Scientific Procedures) Act 1986.

### Fear Conditioning

For all of the experiments background contextual fear conditioning was used. Background contextual fear conditioning involves the hippocampus more strongly than conditioning without a tone presentation [32]. Background conditioning took place in a conditioning chamber (Med Associates Inc, St Albans, USA) that was situated in a soundproof box. The conditioning chamber floor was made up of 36 stainless steel rods that were used for shock delivery. A speaker was mounted on one side of the chamber for delivery of the tone (80 dB, 3.0 kHz). In order to camouflage any noise in the behavioural room background noise was supplied to the chamber by a white noise generator positioned in the side of the soundproof box. Prior to training and contextual fear memory test the chamber was cleaned with 70% ethanol and a paper towel soaked in the ethanol was placed under the grid floor. The cued fear memory test was conducted in a novel chamber that was structurally different from the conditioning chamber. This chamber was semi-circular, had a plastic floor and prior to test, the chamber was cleaned with a lemon-scented solution.

On the conditioning day, the mice were brought from the housing room into a holding room where they were allowed to acclimatise for 30 min before training. During training, the mice were placed individually in the chamber and after a 120 s introductory period a tone (80 dB, 3.0 kHz) was presented for 30 s, the last 2 s of which coincided with a foot shock (0.7 mA). Depending on the intensity of the training protocol the mice received a further 2 or 4 tone-shock pairings at 60 s intervals, and for spaced training they received two training trials with a single tone-shock pairing and an inter-trial interval of 1 h. After training mice were returned to their home cage.

In order to test for short-term contextual memory mice were re-exposed to the conditioning chamber for a period of 5 min 2 h after training. Long-term contextual fear memory was assessed by re-exposing the mice to the conditioning chamber for 5 min 24 h after training. Long-term cued fear memory was assessed by placing each mouse in a novel chamber 48 h after training. Following 180 s without a tone (Pre-CS) the tone was presented for 180 s (CS) to assess cued fear memory. In order to test for the stability of the contextual and cued fear memories the mice were re-tested 30 and 31 days respectively after training.

The behaviour of the mice was recorded and freezing behaviour (defined as complete lack of movement, except for respiration) was scored for 2 s in every 5 s.

### Hippocampal lesions

In order to test if the contextual fear memory after 5 tone-shock pairings was hippocampus-dependent dorsal hippocampal lesions were given to the homozygous αCaMKII^T286A ^mutants and their WT littermates 24 h after training. The mice were anaesthetized with a mixture of isoflourane (Abbott, Kent, UK) and O_2 _and then mounted in a Kopf stereotaxic frame. The scalp was shaved and surgically cleaned and then a midline incision was made that exposed the skull. The skull overlying the target area was removed and bilateral injections of ibotenic acid (Sigma) dissolved in phosphate buffer saline (PBS) at a concentration of 10 mg/ml (see Table 1 for coordinates and volumes; according to [33]) were made using a 5 μl Hamilton syringe with a 33 gauge needle. After completion of the injections, the scalp was sutured, and the mouse was returned to its home cage. Sham-lesioned mice were subjected to the same surgical procedure, but PBS was injected into only one of the dorsal hippocampal coordinates. The mice were tested for contextual fear memory 7 days after surgery.

**Table 1:** Stereotaxic coordinates for lesioning the dorsal hippocampus.

AP (mm)	ML (mm)	DV (mm)	Vol (μl)
- 1.5	1.00	- 1.80	0.07
- 1.8	1.00	- 1.80	0.07
- 1.8	1.75	- 2.00	0.07
- 2.1	1.20	- 1.75	0.07
- 2.1	2.00	- 2.00	0.07
- 2.4	1.30	- 1.75	0.07
- 2.4	2.70	- 2.00	0.07

### Histology

After completion of behavioural testing all lesioned and sham-lesioned mice were given a lethal injection of sodium pentobarbitone (Euthatal; Animal Care Ltd, York, UK) and were perfused with physiological saline and 4% paraformaldehyde in PBS. The brains were removed and stored in 4% paraformaldehyde until they were coronally sectioned at 40 μm and stained with cresyl violet.

### Slice electrophysiology

Hippocampal slices were perfused with artificial cerebrospinal fluid (ACSF) with the following composition: 124 mM NaCl, 5 mM KCl, 26 mM NaHCO_3_, 1.24 mM NaH_2_PO_4_, 2.5 mM CaCl_2_, 1.3 mM MgSO_4_, 10 mM D-glucose, bubbled with a mixture of 95% O_2 _and 5% CO_2_. Mice were anesthetized and decapitated. The hippocampus was dissected and cut in 450 μm-thick slices with a tissue chopper. The slices were transferred into the recording chamber and kept in interface at 28°C for 1.5 h. The perfusion rate of ACSF was 1 ml/min. Bipolar twisted nickel-chrome electrodes (50 μm each) were used to stimulate Schaffer's collaterals.

Extracellular field excitatory postsynaptic potentials (fEPSP) were recorded in the stratum radiatum of the CA1 region with low resistance (2-5 megaOhm) glass microelectrodes filled with ACSF. Test stimuli were biphasic (0.08 ms for each pulse) constant-voltage pulses delivered every minute with an intensity adjusted to evoke an approximate 40% maximal response. The slope of the fEPSP was measured on the average of four consecutive responses. For each slice, an input-output curve was established from the responses obtained for various stimuli. LTP was induced by applying four trains (50 Hz, 1s, 5 min interval). In both protocols, the potentiated response was recorded for 4 h.

For each slice, the fEPSP slopes were normalized with respect to the mean slope of the fEPSPs recorded during the 30-minute period, preceding induction of LTP. To determine whether or not the normalized fEPSP of a group of slices submitted to the same experimental conditions was significantly potentiated (p < 0.05), the percentages of baseline measured after induction of LTP were compared by a two-way ANOVA and several student t tests at different times after LTP induction.

### Statistical analysis

Normally distributed data were analyzed by analysis of variance (ANOVA) followed by Student-Newman-Keuls post-hoc analysis. Not normally distributed data were analyzed by ANOVA on ranks.

## List of abbreviations

αCaMKII: alpha-isoform of the calcium/calmodulin-dependent kinase II; LTM: long-term memory; LTP: long-term potentiation; STM: short-term memory;

## Competing interests

The authors declare that they have no competing interests.

## Authors' contributions

EEI, CN and WL bred and genotyped the αCaMKII^T286A ^mutants. EEI performed the behavioral studies with the mice. AD and LR performed the electrophysiological recordings. EEI, KR, EG, LR and KPG designed the experiments and they wrote the paper. All authors have read and approved the final version of this manuscript.
